# Supplementary material for: Moonlighting chaperone activity of the enzyme PqsE contributes to RhlR-controlled virulence of Pseudomonas aeruginosa
Source: Nat Commun. 2022 Dec 1;13:7402. doi: 10.1038/s41467-022-35030-w (PMC9715718; doi:10.1038/s41467-022-35030-w)

SUPPLEMENTARY INFORMATION

Moonlighting chaperone activity of the enzyme PqsE contributes to RhlR-controlled virulence of *Pseudomonas aeruginosa*

Sebastian Roman Borgert[1,*], Steffi Henke[1,*], Florian Witzgall[1], Stefan Schmelz[1], Susanne zur Lage[1], Sven-Kevin Hotop[2], Steffi Stephen[2], Dennis Lübken[3], Jonas Krüger[4], Nicolas Oswaldo Gomez[4], Marco van Ham[5], Lothar Jänsch[5], Markus Kalesse[3], Andreas Pich[6], Mark Brönstrup[2], Susanne Häussler[4], & Wulf Blankenfeldt[1,7]*

[1]Department Structure and Function of Proteins, Helmholtz Centre for Infection Research, Inhoffenstr. 7, 38124 Braunschweig, Germany

[2]Department Chemical Biology, Helmholtz Centre for Infection Research, Inhoffenstr. 7, 38124 Braunschweig, Germany

[3]Institute of Organic Chemistry, Leibniz University Hannover, Schneiderberg 1B, 30167 Hannover, Germany

[4]Department Molecular Bacteriology, Helmholtz Centre for Infection Research, Inhoffenstr. 7, 38124 Braunschweig, Germany

[5]Cellular Proteomics, Helmholtz Centre for Infection Research, Inhoffenstr. 7, 38124 Braunschweig, Germany

[6]Institute for Toxicology, Core Facility Proteomics, Hannover Medical School, Carl-Neuberg-Str. 1, 30625 Hannover, Germany

[7]Institute for Biochemistry, Biotechnology and Bioinformatics, Technische Universität Braunschweig, Spielmannstr. 7, 38106 Braunschweig, Germany

[*]These authors contributed equally: Sebastian Roman Borgert, Steffi Henke
Email: wulf.blankenfeldt@helmholtz-hzi.de

SUPPLEMENTARY FIGURES

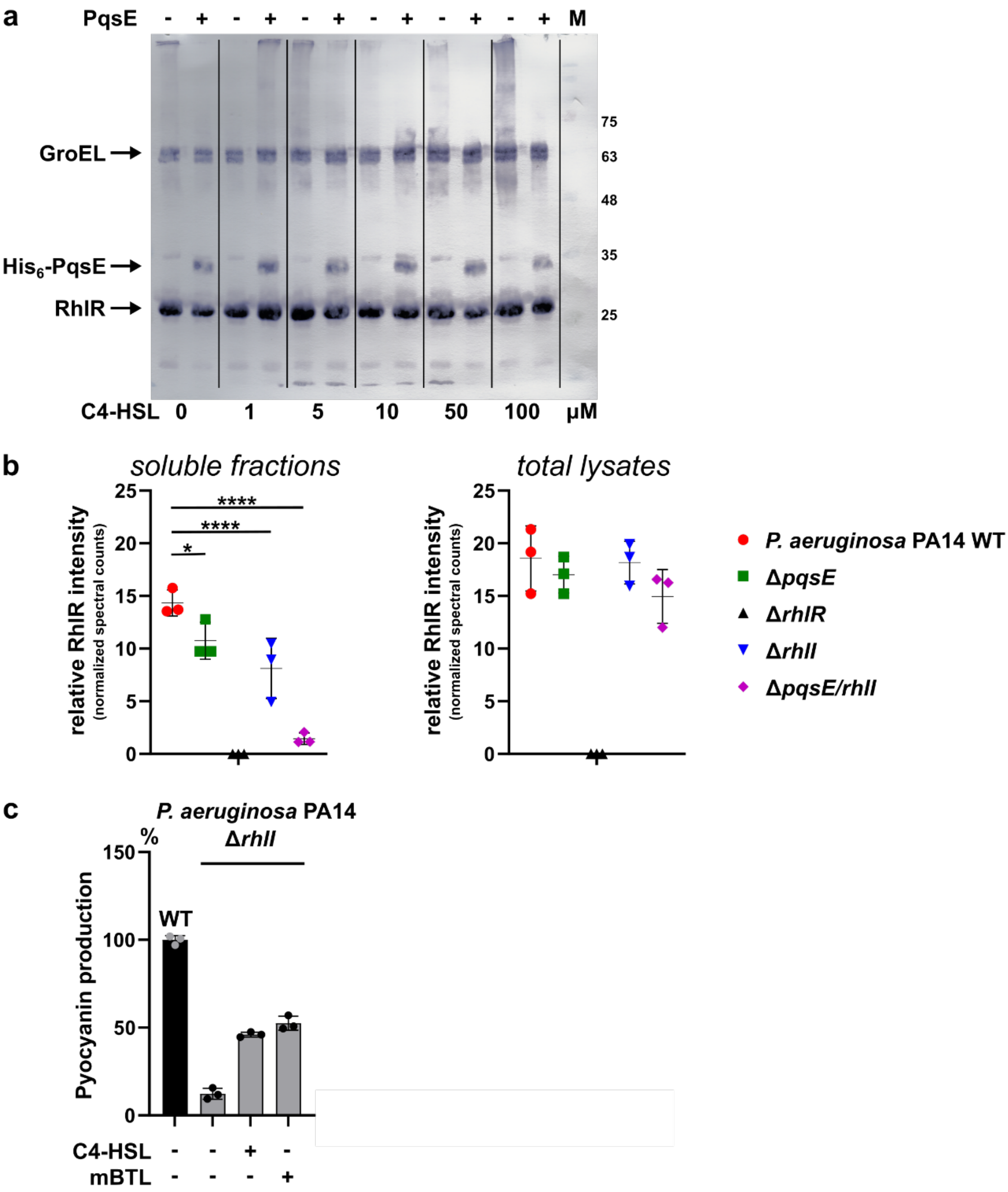

Supplementary Fig. 1 | Impact of C4-HSL, mBTL and PqsE on RhlR and RhlR-mediated pyocyanin production. a Western blot of whole-cell extracts from *E. coli* BL21-CodonPlus(DE3)-RIL overproducing RhlR in the presence of the indicated amounts of C4-HSL and in the presence or absence of simultaneous PqsE-overexpression. The marker (M) indicates the molecular weight in kDa. The total amount of RhlR is not influenced by C4-HSL or PqsE, but the soluble fraction increases with C4-HSL and PqsE (Fig. 2a). **b** Abundance of RhlR in *P. aeruginosa* PA14 and mutants unable to produce RhlR, PqsE and/or RhlI. Dot plots demonstrate that PqsE and RhlI (C4-HSL autoinducer synthase) together increase the amount of soluble RhlR (left), whereas the total amount of RhlR in whole-cell extracts is constant. Relative protein abundances are given in normalized spectral counts as calculated by using the ID_STAT©COMPARE plug-in in PaSER Version 2022c. Statistics: Two-sided Student's t-test: * = p<0.05 (p=0.0449), **** = p<0.0001. Mean values with error bars indicating the standard deviation of three independent measurements are shown. **c** C4-HSL and mBTL lead to similar levels of pyocyanin production in a Δ*rhlI* mutant of *P. aeruginosa* PA14. C4-HSL and mBTL

were applied at a concentration of 50 µM. Mean values with error bars indicating the standard deviation of three independent measurements are shown.

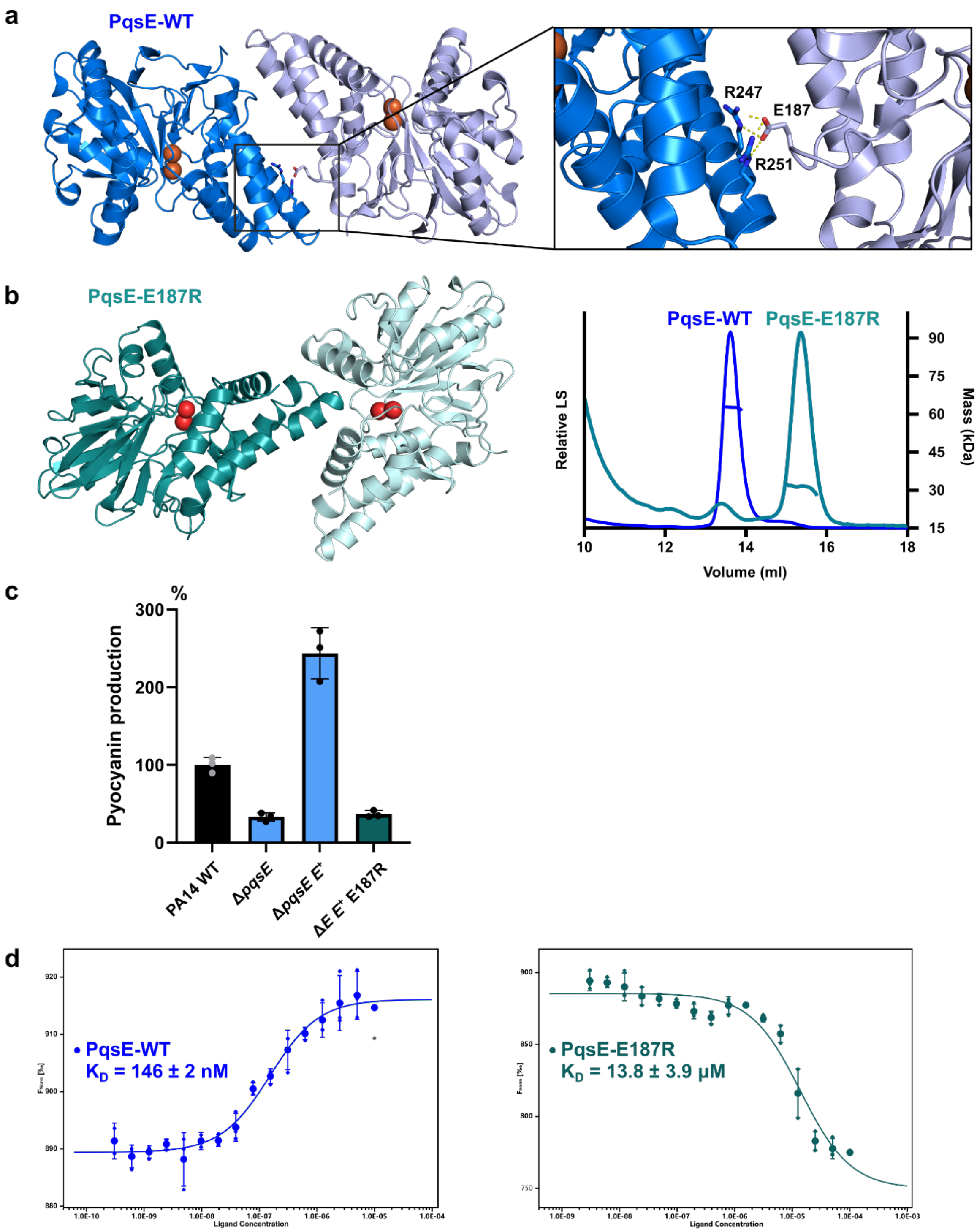

Supplementary Fig. 2 | Dimerization of PqsE impacts on interaction with RhlR and control of the PqsE/RhlR regulon. a Dimers are observed in all crystal structures of WT-PqsE (exemplified with PDB entry 2Q0I[19]). Dimerization involves interactions of E187 with R247 and R251. **b** Mutation of E187 to arginine leads to monomer formation, as demonstrated by crystal structure and SEC-MALS analysis. **c** Monomeric PqsE-E187R does not restore pyocyanin production in a Δ*pqsE*-mutant of *P. aeruginosa* PA14. Mean values with error bars indicating the standard deviation of three independent measurements are shown. **d** Microscalar thermophoresis of PqsE with ATTO488-labeled RhlR:mBTL, revealing a K_D of 146 ± 2 nM (left), whereas the interaction was approx. 100-fold weaker in similar experiments with PqsE-E187R (K_D = 13.8 ± 3.9 µM, right). Mean values with error bars indicating the standard deviation of three independent measurements are shown. Note that PqsE-E187R yielded an inverse titration curve with respect to WT-PqsE.

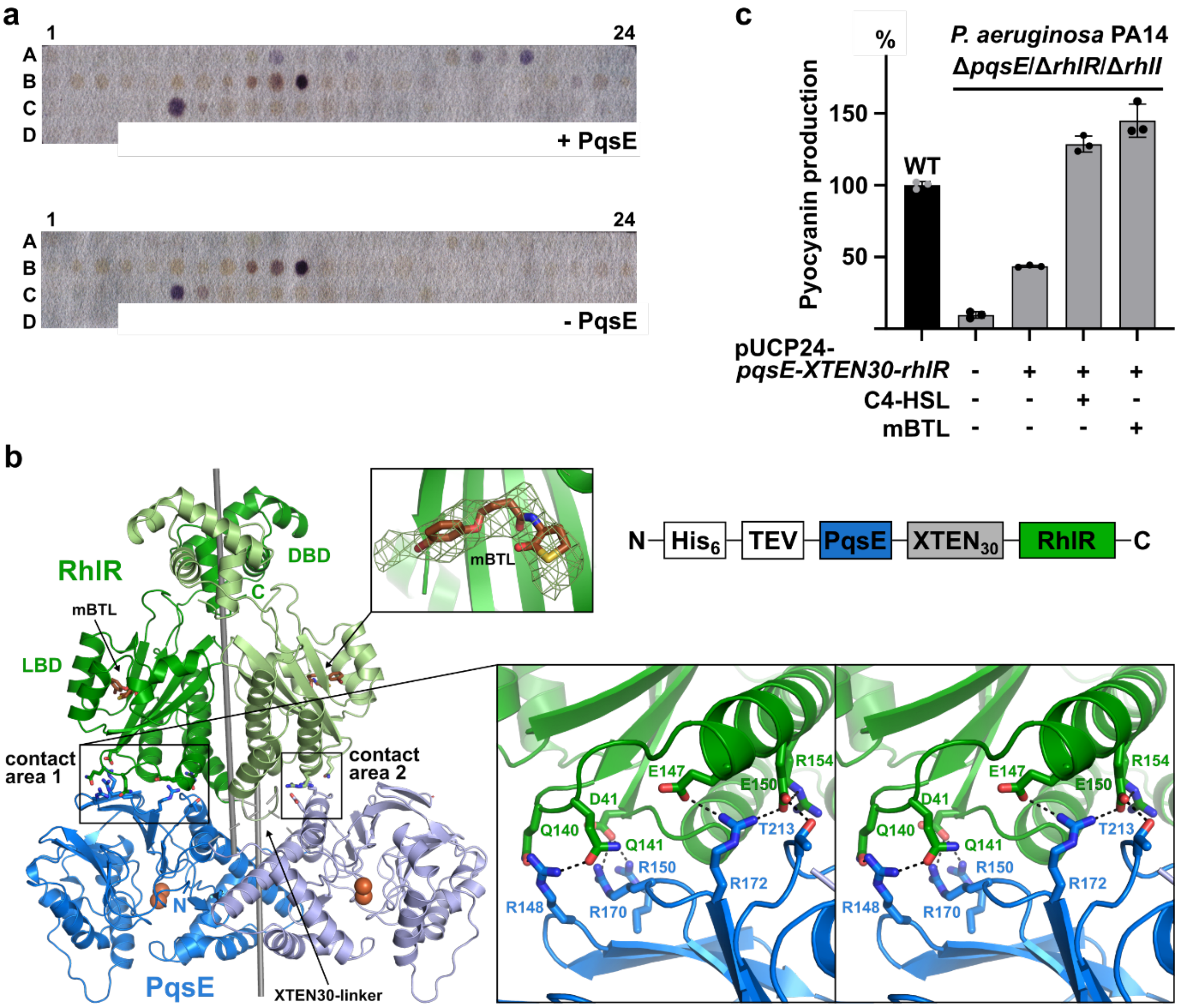

Supplementary Fig. 3 │ Towards structural details of the PqsE/RhlR interaction. a Peptide SPOT array of 21-mer peptides from RhlR, covering the protein sequence in three-residue steps. Peptide sequences are reported in supplementary Tab. 9. The array was incubated with His_6-tagged PqsE and stained with an AP-conjugated anti-His_6 antibody and BCIP. Several specific interactions of PqsE with RhlR-derived peptides can be discerned (upper panel), despite high background found in the negative control (lower panel). b A PqsE-XTEN30-RhlR fusion protein was constructed (schematic overview: upper right; TEV denotes a cleavage site for Tobacco Etch Virus protease) and crystallized in the presence of mBTL. The smaller magnified insert shows $|F_O\text{-}F_C|$ electron density in the ligand binding domain of PqsE-XTEN30-RhlR at 3 σ before incorporating mBTL in the refinement. The larger magnified area shows a cross-eyed stereo plot of polar interactions in the larger contact area between RhlR and PqsE. DBD and LBD denote the DNA- and ligand-binding domains of RhlR, the two-fold symmetry axes of RhlR and PqsE are shown as sticks (calculated with draw_rotation_axis.py in PyMOL[56]). **c** PqsE-XTEN30-RhlR emulates the regulatory interplay of RhlR and PqsE in pyocyanin production as shown in a *P. aeruginosa* PA14 Δ*rhlR*/*rhlI*/*pqsE* triple mutant supplemented with a pUCP24 plasmid encoding the fusion protein. Note that C4-HSL and mBTL stimulate pyocyanin production to similar levels. Mean values with error bars indicating the standard deviation of three independent measurements are shown.

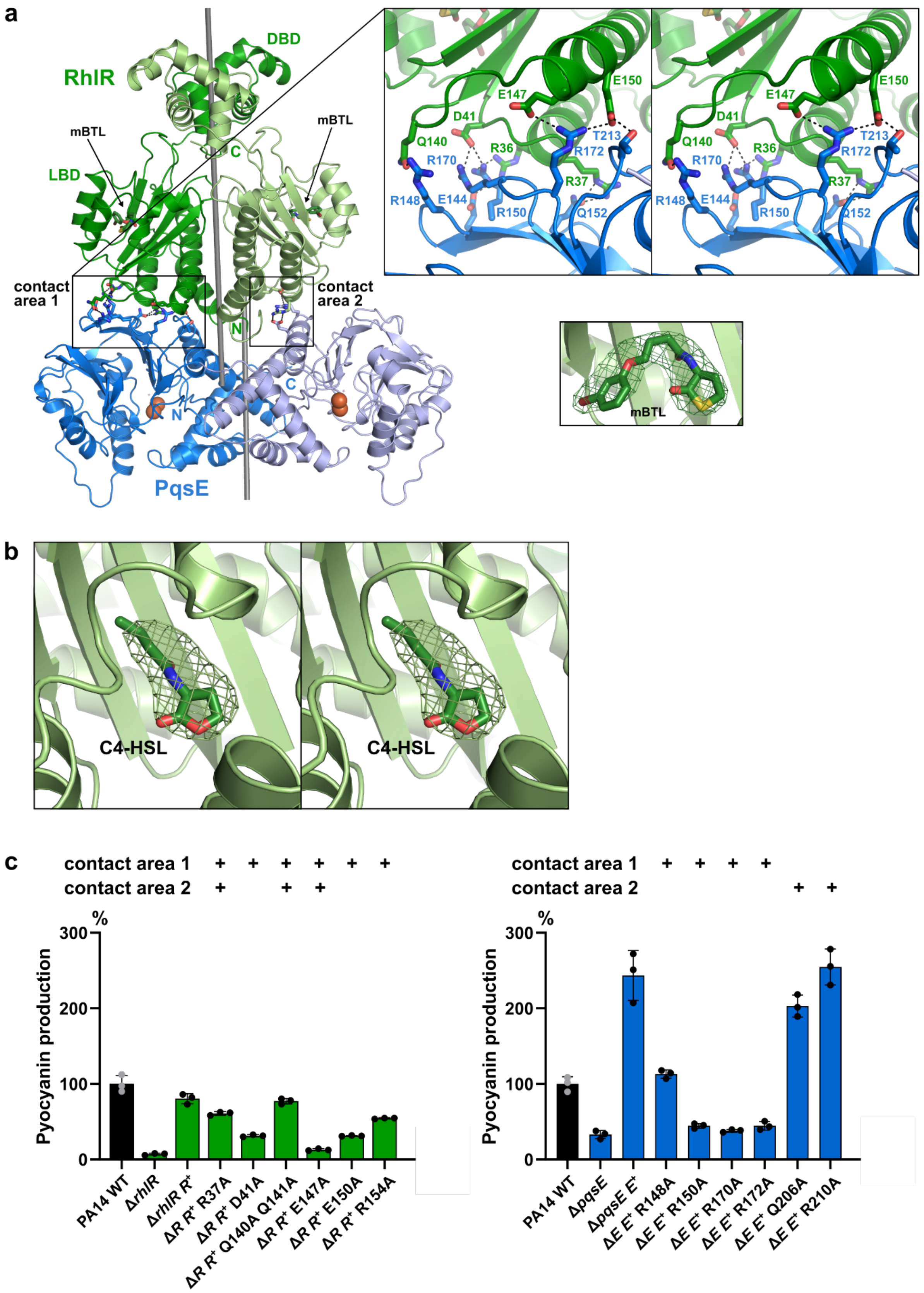

Supplementary Fig. 4 │ Details of the PqsE/RhlR complex structure. a Crystal structure of the PqsE/RhlR:mBTL complex. DBD and LBD denote the DNA- and ligand-binding domains of RhlR, the two-fold symmetry axes of RhlR and PqsE are shown as sticks (calculated with draw_rotation_axis.py in PyMOL[56]). Red spheres represent two iron atoms bound to the active center of PqsE. The smaller magnified insert shows $|F_O\text{-}F_C|$ electron density in the ligand binding domain at 3σ before

incorporating mBTL in the refinement. The larger magnified area shows a cross-eyed stereo plot of polar interactions in the larger contact area between RhlR and PqsE. DBD and LBD denote the DNA- and ligand-binding domains of RhlR. **b** Cross-eyed stereo plot of $|F_O\text{-}F_C|$ electron density at 2.5 σ in the ligand binding domain of the PqsE/RhlR:C4-HSL complex before incorporating the ligand in the refinement. More structural details of this structure are shown in Fig. 3a. **c** Mutagenesis reveals the importance of residues in the larger contact area 1 of the PqsE/RhlR complex for pyocyanin production in *P. aeruginosa* PA14. Contact areas have been analyzed with PISA[57]. Note that contact areas 1 and 2 overlap for RhlR but not for PqsE. Mean values with error bars indicating the standard deviation of three independent measurements are shown.

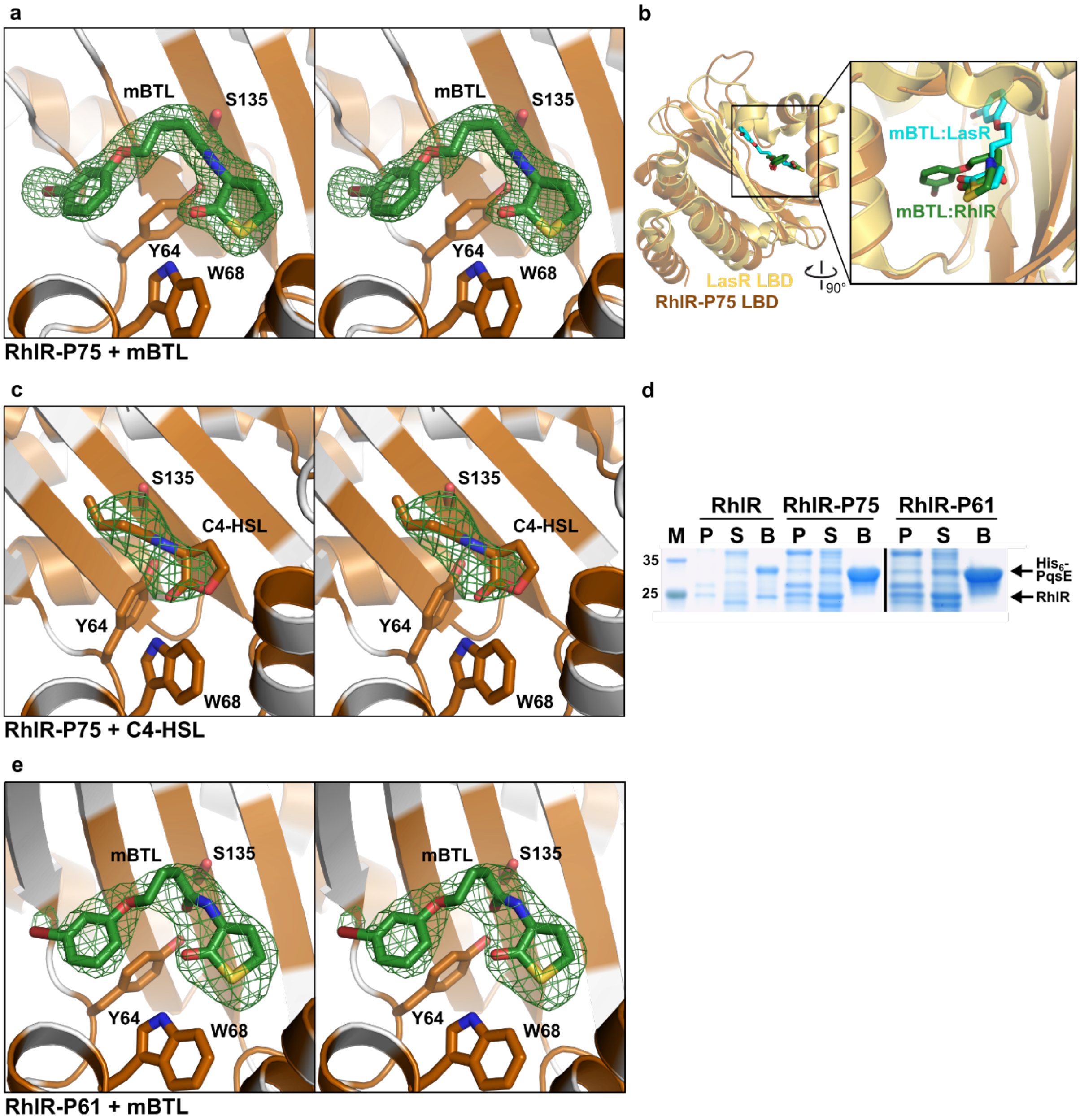

Supplementary Fig. 5 │ Details of crystal structures of RhlR-P75 in complex with mBTL or C4-HSL. a $|F_O\text{-}F_C|$ electron density at 3 σ in the ligand binding domain RhlR-P75 crystallized in the presence of mBTL before incorporating the ligand in the refinement. **b** Overlay of the LBD of RhlR-P75 with the LBD of LasR (PDB entry 6MWL[44]) reveals different conformations of mBTL. **c** $|F_O\text{-}F_C|$ electron density at 2 σ in the ligand binding domain RhlR-P75 crystallized in the presence of C4-HSL before incorporating the ligand in the refinement. **d** RhlR-P75 and RhlR-P61 do not interact with PqsE. SDS-PAGE gel of a co-expression analysis of His_6-tagged PqsE and RhlR-WT, RhlR-P75 or RhlR-P61 in *E. coli* BL21-CodonPlus(DE3)-RIL in the presence of 50 µM C4-HSL. Induced cells were harvested, lysed and separated into an insoluble (P) and a soluble fraction (S), which was used to incubate Ni-NTA beads, washed and then boiled in SDS-loading buffer (B). A typical result from at least three independent experiments is shown. The marker (M) indicates the molecular weight in kDa. **e** $|F_O\text{-}F_C|$ electron density at 3 σ in the ligand binding domain RhlR-P61 crystallized in the presence of mBTL before incorporating the ligand in the refinement.

SUPPLEMENTARY TABLES

Supplementary Tab. 1: Oligonucleotides used in this study

Oligonucleotide	Sequence 5' → 3'	Purpose
PrM_036_XTEN30-F	ACACCAGAATCAGGTCCAGGGACGAGCACTGAACCTTCTGAAGGATCGGCACCAATGAGGAATGACGGAGGCTTTTTG	Forward PCR Primer to introduce a 30 residue XTEN-Linker between PqsE and RhlR
PrM_037_XTEN30-R	CCTGATTCTGGTGTTGCGGACTCGCTTGTTCCTGGTGTCTCACTTCCAGAGTCCAGAGGCAGCGCCTG	Reverse PCR Primer to introduce a 30 residue XTEN-Linker between PqsE and RhlR
PrM_044_R150A-F	CGGCATGCACTGCAGGTCATAGAGGCCC	Forward SDM Primer to introduce R150A Substition in PqsE
PrM_045_R150A-R	CTGCAGTGCATGCCGCGGTCCCAG	Reverse SDM Primer to introduce R150A Substition in PqsE
PrM_046-R170A-F	GACGTGGCACGCCGCCGCCTGTTCTG	Forward SDM Primer to introduce R170A Substition in PqsE
PrM_047-R170A-R	GCGGCGTGCCACGTCGTAGAAAACCACG	Reverse SDM Primer to introduce R170 Substition in PqsE
PrM_048-R172A-F	CGACGTGCCCGCCTGTTCTGCGGC	Forward SDM Primer to introduce R172A Substition in PqsE
PrM_049-R172A-R	CAGGCGGGCACGTCGCACGTCGTAGAAAACC	Reverse SDM Primer to introduce R172A Substition in PqsE
PrM_050-E206A-F	TCCCTGGCACGTCTGCAGCGTCTGC	Forward SDM Primer to introduce E206A Substition in PqsE
PrM_051-E206A-R	CAGACGTGCCAGGGACTCCAGGTAAGCC	Reverse SDM Primer to introduce E206A Substition in PqsE
PrM_052-Q209A-F	CGTCTGGCACGTCTGCCGACCCTGC	Forward SDM Primer to introduce Q209A Substition in PqsE
PrM_053-Q209A-R	CAGACGTGCCAGACGTTCCAGGGACTCC	Reverse SDM Primer to introduce Q209A Substition in PqsE
PrM_054-R210A-F	CTGCAGGCACTGCCGACCCTGCTGC	Forward SDM Primer to introduce R210A Substition in PqsE
PrM_055-R210A-R	CGGCAGTGCCTGCAGACGTTCCAGGGAC	Reverse SDM Primer to introduce R210A Substition in PqsE
PrM_058-R148A-F	CCGGCACATCGCCTGCAGGTCATAGAG	Forward SDM Primer to introduce R148A Substition in PqsE

PrM_059-R148A-R	CAGGCGATGTGCCGGTCCCAGCTC CAGCC	Reverse SDM Primer to introduce R148A Substition in PqsE
Pr029_PqsE-Duet_F	CTTTAAGAAGGAGATATACCATGG GCCATCATCATCATCATCA	Forward Primer to amplify pqsE sequence
Pr030_PqsE-Duet_R	TACGATTACTTTCTGTTCGATCAGT CCAGAGGCAGCG	Reverse Primer to amplify pqsE sequence
Pr031_Duet_Frag_F	TCGAACAGAAAGTAATCGTATTGT ACACG	Forward Primer to amplify the region between the two MCS in pET-Duet-1 sequence
Pr032_Duet_Frag_R	ATGTATATCTCCTTCTTATACTTAAC TAATATACTAAGATGGGGAATTG	Reverse Primer to amplify the region between the two MCS in pET-Duet-1 sequence
Pr033_RhlR_F	TATAAGAAGGAGATATACATATGA GGAATGACGGAGGCTTTT	Forward Primer to amplify rhlR sequence
Pr034_RhlR_R	TTTACCAGACTCGAGGGTACTCAG ATGAGACCCAGCGCC	Reverse Primer to amplify rhlR sequence
Pr039_RhlR_500-F	CGGGGATCCTCTAGAGTCGACGCA GCGCGCCTACGC	Forward Primer to amplify the 500 bp upstream region of rhlR
Pr041-RhlR-Prom-R	CATTGCAGTAAGCCCTGATCGATAA AATGCATC	Reverse Primer to amplify the 500 bp upstream region of rhlR
Pr042-RhlR-F	ATCGATCAGGGCTTACTGCAATGA GGAATGACGGAGGCTTTTTG	Forward Primer to amplify the rhlR sequence for cloning in pUCP24m
Pr036_His_RhlR_R	ACGACGGCCAGTGCCAAGCTTCAG ATGAGACCCAGCGCC	Reverse Primer to amplify the rhlR sequence for cloning in pUCP24m
Pr043-RhlR-75-F	GATCAGGGCTTACTGCAATGCGCA ACGATGGCGGCTTTC	Forward Primer to amplify the rhlR-PROSS mutant sequences for cloning in pUCP24m
Pr038_His_RhlR_P75_R	AAACGACGGCCAGTGCCAAGCTTA AATCAGGCCCAGCGCCG	Reverse Primer to amplify the rhlR-PROSS mutant sequences for cloning in pUCP24m
PqsE_E187R_for	GGGCGAGTTCGACGAGGCACGTG G GGTGTGGCGGCCGCTGG	Forward QuikChange Primer to introduce the named mutations into PqsE
PqsE_E187R_rev	CCAGCGGCCGCCACACCCCAC GTGCCTCGTCGAACTCGCCC	Reverse QuikChange Primer to introduce the named mutations into PqsE
RhlRwt_PstI	AAACTGCAGTCCAACCCGGTCTGCC TGA	Forward PCR Primer to amplify RhlR WT DBD with PstI restriction site
RhlRwt_KpnI_rv	AAAGGTACCTCAGATGAGACCCAG CGCCGC	Reverse PCR Primer to amplify RhlR WT DBD with KpnI restriction site

RP75_S173MQ175M_fw	CGAACTGAACCATCCGATGCTGATGTCCAACCCGGTCTGCC	Forward QuikChange Primer to introduce the named mutations into RhlR-P75
RP75_S173MQ175M_rv	GGCAGACCGGGTTGGACATCAGCATCGGATGGTTCAGTTCG	Reverse QuikChange Primer to introduce the named mutations into RhlR-P75
RhlR_WT_R37A_fw	GGAAAAGGAAGTGCGGGCCCTGGGCTTCGATTAC	Forward QuikChange Primer to introduce the named mutation into RhlR WT
RhlR_WT_R37A_rv	GTAATCGAAGCCCAGGGCCCGCACTTCCTTTTCC	Reverse QuikChange Primer to introduce the named mutation into RhlR WT
RhlR_WT_D41A_fw	GCGCCTGGGCTTCGCTTACTACGCCTATG	Forward QuikChange Primer to introduce the named mutation into RhlR WT
RhlR_WT_D41A_rv	CATAGGCGTAGTAAGCGAAGCCCAGGCGC	Reverse QuikChange Primer to introduce the named mutation into RhlR WT
RhlR_WT_E147A_fw	CATCTCCAGCTTCGCGCGCGAGGAAATCC	Forward QuikChange Primer to introduce the named mutation into RhlR WT
RhlR_WT_E147A_rv	GGATTTCCTCGCGCGCGAAGCTGGAGATG	Reverse QuikChange Primer to introduce the named mutation into RhlR WT
RhlR_WT_E150A_fw	TCGAGCGCGAGGCAATCCGCCTGCG	Forward QuikChange Primer to introduce the named mutation into RhlR WT
RhlR_WT_E150A_rv	CGCAGGCGGATTGCCTCGCGCTCGA	Reverse QuikChange Primer to introduce the named mutation into RhlR WT
RhlR_WT_R36A_fw	TGGAAAAGGAAGTGGCGCGCCTGGGCTTCG	Forward QuikChange Primer to introduce the named mutation into RhlR WT
RhlR_WT_R36A_rv	CGAAGCCCAGGCGCGCCACTTCCTTTTCCA	Reverse QuikChange Primer to introduce the named mutation into RhlR WT
RhlR_WT_Q140AQ141A_fw	TTTCCGTGGCGCGCGACGCGGCGAACATCTCCAGCTTCG	Forward QuikChange Primer to introduce the named mutations into RhlR WT
RhlR_WT_Q140AQ141A_rv	CGAAGCTGGAGATGTTCGCCGCGTCGCGCGCCACGGAAA	Reverse QuikChange Primer to introduce the named mutations into RhlR WT
RWT_R154A_fw	GAGGAAATCCGCCTGGCGCTGCGTTGCATGA	Forward QuikChange Primer to introduce the named mutation into RhlR WT
RWT_R154A_rv	TCATGCAACGCAGCGCCAGGCGGATTTCCTC	Reverse QuikChange Primer to introduce the named mutation into RhlR WT
*pqsE*_up_F	CGAATTCGGCGAGAGTCTCGAAGACG	Forward PCR primer for the amplification of upstream

		pqsE sequence (EcoRI site underlined)
*pqsE*_up_R	CAGAGGCAGCGAAAGCCTCAACATGGC	Reverse PCR primer for the amplification of upstream *pqsE* sequence
*pqsE*_dw_F	AGGCTTTCGCTGCCTCTGGACTGAGGC	Forward PCR primer for the amplification of downstream *pqsE* sequence
*pqsE*_dw_R	CGGATCCCTGCGACTCTTCCTGGGG	Reverse PCR primer for the amplification of downstream *pqsE* sequence (BamHI site underlined)
pqsE_F	CAACCTGTCGATCCTGCTCG	Forward primer for the verification of the *pqsE* deletion
pqsE_R	CAGGCTGGACAGGCCATG	Reverse primer for the verification of the *pqsE* deletion
rhlR_Up_F	TATGCGGCCGCTGCAGCGCGCCTACGCG	Forward PCR primer for the amplification of upstream *rhlR* sequence (NotI site underlined)
rhlR_Up_R	TCAGTCAGTCAGCTAGCTGCAGTAAGCCCTGATCGATAAAATGCA	Reverse PCR primer for the amplification of upstream *rhlR* sequence (NheI site underlined)
rhlR_Dw_F	GCTAGCTGACTGACTGAAGCGCAGGGCGCGCCG	Forward PCR primer for the amplification of upstream *rhlR* sequence (NheI site underlined)
rhlR_Dw_R	TATAAGCTTGGCGGCGTAGCGCGAAAGC	Reverse PCR primer for the amplification of downstream *rhlR* sequence (HindIII site underlined)
*rhlR*_F	CTGTCGGCGTTTCATGGAATTG	Forward primer for the verification of the *rhlR* deletion
*rhlR*_R	AAGACTTGATGCCGGTAGCGTC	Reverse primer for the verification of the *rhlR* deletion
Sp_*rhlI*_F	gcgcgCGAATTGCTCTCTGAATCGC	Sense-oriented oligonucleotide to construct the specific *rhlI* spacer (protruding end indicated in lowercase)
Sp_*rhlI*_R	aaacGCGATTCAGAGAGCAATTCGc	Antisense-oriented oligonucleotide to construct the specific *rhlI* spacer (protruding end indicated in lowercase)

*rhlI*_ ssDNA_R	CATGCGGCAGGAGAAGCGGAAAAAAGTGCGCGAAACGGCTGACGACC*TCA***CAT**GACCAAGTCCCCGTGTCGTGCCGGCCGAGAAAAAAAAGGCGGCATCC	Recombineeting mutagenic oligonucleotide to generate the clean deletion of *rhlI* (Start codon bold and Stop codon in italic)
*rhlI*_F	TCCTCCTTTAGTCTTCCCCC	Forward primer for the verification of the *rhlI* deletion
*rhlI*_R	GAGAGACTACGCAAGTCGGC	Reverse primer for the verification of the *rhlI* deletion

Primers were ordered from either Eurofins Genomics or Sigma Aldrich.

Supplementary Tab. 2: Plasmids used in this study

Plasmid	Features / Function	Supplier / Source
pET-Duet-1		Novagen
pUCP24m	pUCP24 Vector with an introduced stop codon in the lacZα-peptide	kindly provided by Prof. D. Jahn
pUCP24m His-PqsE WT	pUCP24m derivative encoding the sequence for PqsE with a N-terminal His-Tag	this study
pUCP24m His-PqsE-R148A	pUCP24m derivative encoding the sequence for PqsE-R148A with a N-terminal His-Tag	this study
pUCP24m His-PqsE-R150A	pUCP24m derivative encoding the sequence for PqsE-R150A with a N-terminal His-Tag	this study
pUCP24m His-PqsE-R170A	pUCP24m derivative encoding the sequence for PqsE-R170A with a N-terminal His-Tag	this study
pUCP24m His-PqsE-R172A	pUCP24m derivative encoding the sequence for PqsE-R172A with a N-terminal His-Tag	this study
pUCP24m His-PqsE-E187R	pUCP24m derivative encoding the sequence for PqsE-E187R with a N-terminal His-Tag	this study
pUCP24m His-PqsE-E206A	pUCP24m derivative encoding the sequence for PqsE-E206A with a N-terminal His-Tag	this study
pUCP24m His-PqsE-Q209A	pUCP24m derivative encoding the sequence for PqsE-Q209A with a N-terminal His-Tag	this study
pUCP24m His-PqsE-R210A	pUCP24m derivative encoding the sequence for PqsE-R210A with a N-terminal His-Tag	this study
pUCP24m His-PqsE-XTEN30-RhlR	pUCP24m derivative encoding the sequence for PqsE and RhlR linked by a 30 residue XTEN Linker and a N-terminal His-Tag	this study
pUCP24m prhlR RhlR WT	pUCP24m derivative encoding the wildtype RhlR sequence and 500 bp of the region upstream of RhlR	this study
pUCP24m prhlR RhlR R37A	pUCP24m derivative encoding the RhlR-R37A sequence and 500 bp of the region upstream of RhlR	this study
pUCP24m prhlR RhlR D41A	pUCP24m derivative encoding the RhlR-D41A sequence and 500 bp of the region upstream of RhlR	this study
pUCP24m prhlR RhlR E147A	pUCP24m derivative encoding the RhlR-E147A sequence and	this study

	500 bp of the region upstream of RhlR	
pUCP24m prhlR RhlR E150A	pUCP24m derivative encoding the RhlR-E150A sequence and 500 bp of the region upstream of RhlR	this study
pUCP24m prhlR RhlR R154A	pUCP24m derivative encoding the RhlR-R154A sequence and 500 bp of the region upstream of RhlR	this study
pUCP24m prhlR RhlR Q140A Q141A	pUCP24m derivative encoding the RhlR-Q140A Q141A sequence and 500 bp of the region upstream of RhlR	this study
pUCP24m prhlR RhlR P61	pUCP24m derivative encoding the RhlR-P61 sequence and 500 bp of the region upstream of RhlR	this study
pVP008 Strep-TEV-RhlR-P75	pCOLA Duet (Novagen) derivate with TEV protease cleavage site, RhlR-P75 cloned between NotI/KpnI restriction sites	this study
pVP008 Strep-TEV-RhlR P61	pCOLA Duet (Novagen) derivate with TEV protease cleavage site, RhlR-P61 cloned between NotI/KpnI restriction sites	this study
pET19m His_6-TEV-PqsE	pET19b (Novagen) derivate with TEV protease cleavage site and sequence encoding for PqsE	Zender and Witzgall et al. 2016
pET19m His_6-TEV-PqsE-E187R	pET19b (Novagen) derivate with TEV protease cleavage site and sequence encoding for PqsE-E187R	this study
pET-Duet-1 His-TEV-PqsE x RhlR	pET-Duet-1 (Novagen) derivate with TEV protease cleavage site and sequence encoding for PqsE (MCS1) and RhlR (MCS2)	this study
pET-Duet-1 His-TEV-PqsE-XTEN30-RhlR	pET-Duet-1 (Novagen) derivate with TEV protease cleavage site and sequence encoding for PqsE and RhlR linked by a 30 residue XTEN linker	this study
pET26b RhlR WT	Expression vector for untagged RhlR WT, RhlR-encoding sequence cloned between NdeI/XhoI restriction sites	this study
pET19m His_6-TEV-RhlR WT	pET19b (Novagen) derivate with TEV protease cleavage site and sequence encoding for RhlR	this study

pS448•CsR	Sm^{R}, *oriV pRO1600/ColE1*, *oriT*, *xyl*S, *Pm*□*cas*9, P_{EM7}□sgRNA	Seupt et al.
pSEVA624-*ssr*	Gm^{R}, pSEVA624 cloned with the *ssr* recombinase sequence	Seupt et al.
pEX-pqsE	Gene replacement vector for PA14 *pqsE*, Amp^{R}	This study
pEX18Ap2-*rhlR*	Gene replacement vector for PA14 *rhlR* containing a FRT-Gm cassette, Amp^{R}	This study
pFLP3	FLP expression vector, sacB*, oriT^{+}, Ap^{R} Tet^{R}	Choi, Gaynor et al. 2005
pS448•CsR-*rhlI*	Sm^{R}, derivative of pS448•CsR carrying a P_{EM7}□*rhl*I-targeting sgRNA	This study

Supplementary Tab. 3: Strains used in this study

Strain	Features / Genotype	Supplier / Source
Escherichia coli Omnimax 2	F´ {*pro*AB *lac*I^{q} *lac*ZΔM15 *Tn*10(Tet^{R}) Δ(*ccd*AB)} *mcr*A Δ(*mrr hsd*RMS-*mcr*BC) Φ80(*lac*Z)ΔM15 Δ(*lac*ZYA-*arg*F)U169 *end*A1 *rec*A1 *sup*E44 *thi*-1 *gyr*A96 *rel*A1 *ton*A *pan*D	Invitrogen
Escherichia coli BL21 Codon+ RIL	*F´ ompT hsdS(rB– mB –) dcm+ Tet^{R} gal λ(DE3) endA Hte [argU ileY leuW Cam^{R}]*	Agilent
Pseudomonas aeruginosa PA14	Wildtype	Liberati, Urbach *et al*. 2006
Pseudomonas aeruginosa PA14 *ΔrhlR*	Knockout strain *ΔrhlR*	Seupt *et al*.
Pseudomonas aeruginosa PA14 *ΔpqsE*	Knockout strain *ΔpqsE*	This study
Pseudomonas aeruginosa PA14 *ΔpqsE ΔrhlR*	Knockout strain *ΔpqsE ΔrhlR*	This study
Pseudomonas aeruginosa PA14 *ΔrhlI*	Knockout strain *ΔrhlI*	This Study
Pseudomonas aeruginosa PA14 *ΔpqsE ΔrhlI ΔrhlR*	Knockout strain *ΔpqsE ΔrhlI ΔrhlR*	This study
Pseudomonas aeruginosa PA14 tn*pqsE* pUCP20::His$_{6}$-*pqsE*	Transposon insertion in *pqsE* transformed with pUCP20::His$_{6}$-*pqsE*	This study
Pseudomonas aeruginosa PA14 Δ20700ΩGm pUCP20::His$_{6}$-*flgZ*	Transposon insertion inf flgZ transformed with pUCP20::His$_{6}$-*flgZ*	This study

Supplementary Tab. 4: Main buffers used in this study

**Buffer**	
Lysis Buffer RhlR-P75/P61	50 mM Tris pH 8.0, 500 mM NaCl, 5 mM DTT
Lysis Buffer RhlR WT	50 mM Tris-HCl pH 8.0, 500 mM NaCl, 1 mM DTT, 5% glycerol, 50 µM mBTL
Lysis Buffer XTEN	50 mM Bicine pH 8.0, 150 mM NaCl, 5% glycerol, 50 µM mBTL
Lysis Buffer PqsE/RhlR-complex	50 mM Bicine pH 8.0, 150 mM NaCl, 5% glycerol, 20 µM C4-HSL
Lysis Buffer PqsE/RhlR-complex WB	50 mM Bicine pH 8.0, 150 mM NaCl, 50 µM C4-HSL
Lysis Buffer PqsE E187R	50 mM Tris pH 8.0, 150 mM NaCl, 10% glycerol
Elution buffer RhlR-P75/P61	50 mM Tris-HCl pH 8.0, 500 mM NaCl, 5 mM DTT, 5 mM d-desthiobiotin
Elution Buffer RhlR WT	50 mM Tris-HCl pH 8.0, 500 mM NaCl, 50 µM mBTL, 500 mM imidazole
Elution buffer XTEN	50 mM Bicine pH 8.0, 150 mM NaCl, 5% glycerol, 500 mM Imidazol, 50 µM mBTL
Elution buffer PqsE/RhlR-complex	50 mM Bicine pH 8.0, 150 mM NaCl, 5% glycerol, 20 µM C4-HSL, 500 mM Imidazole
Elution Buffer PqsE E187R	50 mM Tris pH 8.0, 150 mM NaCl, 500 mM imidazol, 10% glycerol
Dialysis buffer RhlR-P75/P61	50 mM Tris-HCl pH 8.0, 500 mM NaCl, 5 mM DTT
Dialysis Buffer RhlR WT	50 mM Tris-HCl pH 8.0, 500 mM NaCl, 50 µM mBTL
Ion Exchange buffer PqsE/RhlR-complex	Buffer A: 50 mM Bicine pH 8.0, 5% glycerol, 20 µM C4-HSL<br>Buffer B: 50 mM Bicine pH 8.0, 1 M NaCl, 5% glycerol, 20 µM C4-HSL
SEC buffer RhlR-P75/P61	50 mM Tris-HCl pH 8.0, 500 mM NaCl, 5 mM DTT, 50 µM mBTL
SEC Buffer RhlR WT	50 mM Tris-HCl pH 8.0, 500 mM NaCl, 50 µM mBTL
SEC buffer XTEN	50 mM Bicine pH 8.0, 100 mM NaCl, 5% glycerol, 50 µM mBTL
SEC buffer PqsE/RhlR-complex	50 mM Bicine pH 8.0, 50 mM NaCl, 5% glycerol, 40 µM C4-HSL
SEC Buffer PqsE E187R	50 mM Tris pH 8.0, 150 mM NaCl, 10% glycerol

Supplementary Tab. 5: Antibodies used in this study

**Antibody**	**Description**	**Source**
α-RhlR	anti-RhlR polyclonal rabbit IgG, f.c. 1:33333	Davids Biotechnologie GmbH
α-PqsE	anti-PqsE (residues 256-267) polyclonal rabbit IgG, f.c. 1:5000	BioGenes GmbH
α-groEL	anti-groEL polyclonal rabbit IgG, f.c. 1:10000	Abcam ab90522, Lot GR3394934-8
α-Rabbit-AP	anti-rabbit polyclonal goat IgG (Fc), AP-conjugated, f.c. 1:7500	Promega S3731, Lot 000515550
α-His_6-AP	anti-His6 monoclonal mouse IgG, AP-conjugated, f.c. 1:1000	GeneTex GTX44021, Lot 821602997

Supplementary Tab. 6: Crystallization conditions and cryo-protectants used in this study

	**Temperature**	**Precipitant**	**Protein concentration**	**Cryoprotectant**
**RhlR-P75:mBTL**	293 K	0.1 M Bis-Tris pH 6.5, 0.2 M NaCl, 25 % (w/v) PEG 3350	3.5 mg/ml	10% (v/v) (*2R,3R*) -(-)-2,3-butanediol
**RhlR-P75:C4-HSL**	293 K	0.2 M NaSCN, 20 % (w/v) PEG 3350	3.5 mg/ml	10% (v/v) (*2R,3R*) -(-)-2,3-butanediol
**RhlR-P61:mBTL**	293 K	50 mM BICINE pH 8.8, 8.6 % (w/v) PEG 2K, 17.1 % (v/v) PEG MME 350	3 mg/ml	10% (v/v) (*2R,3R*) -(-)-2,3-butanediol
**PqsE-$XTEN_{30}$-RhlR:mBTL**	293 K	0.1 M NaAcetate pH 4.22, 1.029 M $(NH_4)_2HPO_4$	12 mg/ml	10% (v/v) (*2R,3R*) -(-)-2,3-butanediol
**PqsE/RhlR:C4-HSL**	293 K	1.1 M $(NH_4)_2HPO_4$, 10 mM THPP (pH 7), 100 µM C4-HSL	6 mg/ml	30% (w/v) glycerol
**PqsE/RhlR:mBTL**	293 K	0.1 M NaAcetate pH 4.91, 0.972 M $(NH_4)_2HPO_4$, 10 mM TCEP pH 7	10 mg/ ml	20% (w/v) glycerol
**PqsE-E187R**	293 K	0.16 M CaAcetate, 0.08 M NaCacodylate pH 6.5, 14.4% PEG 8000, 20% glycerol	7.5 mg/ ml	10% (v/v) (*2R,3R*) -(-)-2,3-butanediol

All crystallization experiments were performed in Intelli-Plates (MiTeGen) with crystallization droplets of 200 nl protein and 200 precipitant solution equilibrated against 75 µl reservoirs. PqsE-XTEN30-RhlR:mBTL and PqsE/RhlR:C4-HSL were crystallized in MRC Maxi 48-well crystallization plates with 1 µl protein and 1 µl reservoir or 2 µl protein and 2 µl reservoir solution equilibrated against 100 µl reservoir, respectively.

Supplementary Tab. 7: Data collection statistics

PDB entry	RhlR-P75 (mBTL) 7R3G	RhlR-P75 (C4-HSL) 7R3H	RhlR-P61 (mBTL) 7R3I	PqsE-XTEN30-RhlR (mBTL) 7R3E	PqsE/RhlR complex (mBTL) 7R3J	PqsE/RhlR complex (C4-HSL) 8B4A	PqsE-E187R 7R3F
Space group	$P6_1$	$P2_12_12_1$	$P4_12_12$	I422	I422	I422	$P2_12_12_1$
Cell dimensions							
a, *b*, *c* (Å)	164.75, 164.75, 40.3	87.98, 91.55, 117.37	85.90, 85.90, 202.59	159.65, 159.65, 288.74	159.26, 159.26, 284.10	158.88, 159.88, 285.73	45.53, 60.17, 128.26
α, β, γ (°)	90.0, 90.0, 120.0	90.0, 90.0, 90.0	90.0, 90.0, 90.0	90.0, 90.0, 90.0	90.0, 90.0, 90.0	90.0, 90.0, 90.0	90.0, 90.0, 90.0
Resolution (Å)*	47.57-2.15 (2.23 - 2.15)	72.18-3.49 (3.62-3.49)	79.16-3.1 (3.21 - 3.1)	144.38-3.46 (3.71-3.46)	47.47-3.06 (3.33-3.06)	142.87-3.06 (3.37-3.06)	27.68-1.60 (1.66-1.60)
Max. ellipsoidal resolution (Å)[a] (direction)[b]				3.46 (a*) 3.46 (b*) 4.17 (c*)	3.06 (a*) 3.06 (b*) 3.97 (c*)	3.06 (a*) 3.06 (b*) 3.82 (c*)	
R_{merge}*	0.231 (2.304)	0.385 (1.660)	0.129 (0.846)	0.126 (2.961)	0.184 (2.793)	0.116 (1.590)	0.107 (0.592)
R_{pim}*	0.0399 (0.418)	0.0635 (0.318)	0.0148 (0.132)	0.025 (0.563)	0.036 (0.541)	0.037 (0.497)	0.038 (0.316)
CC(1/2)*	0.998 (0.711)	0.997 (0.785)	1 (0.946)	0.999 (0.562)	0.999 (0.591)	0.999 (0.630)	0.998 (0.681)
I / σ(I)*	11.1 (1.6)	7.6 (2.1)	18.5 (2.5)	17.5 (1.5)	17.0 (1.6)	12.8 (1.8)	13.06 (2.22)
Completeness							
spherical (%)*	100 (100)	100 (100)	100 (100)	79.0 (21.8)	73.8 (24.6)	76.3 (28.0)	99.45 (95.72)
ellipsoidal (%)*				94.8 (58.2)	95.0 (74.2)	94.9 (70.7)	
Redundancy*	20.1 (19.8)	13.0 (13.7)	25.5 (27.2)	26.8 (28.4)	27.2 (27.4)	10.7 (11.2)	8.08 (2.80)

*Values in parentheses are for highest-resolution shell.

[a]Data were processed anisotropically via STARANISO.

[b]Resolution limits for three directions in reciprocal space (a*, b*, c*).

Supplementary Tab. 8: **Refinement statistics**

PDB entry	RhlR-P75 (mBTL) 7R3G	RhlR-P75 (C4-HSL) 7R3H	RhlR-P61 (mBTL) 7R3I	PqsE-XTEN30-RhlR (mBTL) 7R3E	PqsE/RhlR complex (mBTL) 7R3J	PqsE/RhlR complex (C4-HSL) 8B4A	PqsE-E187R 7R3F
Resolution (Å)*	47.57-2.15 (2.22 - 2.15)	49.4-3.49 (3.62 - 3.49)	43.63-3.1 (3.21 - 3.1)	53.54-3.46 (3.71 - 3.46)	47.47 - 3.06 (3.17 - 3.064)	56.52-3.06 (3.18-3.06)	29.29 - 1.65 (1.71 - 1.65)
No. reflections	34603	12536	14454	19554 (ellipsoidal)	25592 (ellipsoidal)	26936 (ellipsoidal)	43230
R_{work}*	0.199 (0.264)	0.223 (0.262)	0.248 (0.296)	0.228 (0.363)	0.269 (0.460)	0.258 (0.414)	0.206 (0.266)
R_{free}*	0.230 (0.307)	0.270 (0.347)	0.272 (0.346)	0.243 (0.405)	0.289 (0.475)	0.278 (0.425)	0.263 (0.291)
No. atoms							
Protein	3780	7278	3577	8610	8623	8598	2339
Ligand/ion	72	50	72	76	76	28	21
Water	136	0	0	3	23	14	264
B-factors							
Protein	46.70	78.47	93.77	175.07	112.63	121.85	16.26
Ligand/ion	33.46	97.95	76.29	163.11	102.93	103.32	20.06
Water	44.32	0	0	178.51	90.71	109.46	24.49
R.m.s. deviations							
Bond lengths (Å)	0.003	0.002	0.002	0.003	0.003	0.002	0.005
Bond angles (°)	0.605	0.487	0.516	0.670	0.613	0.509	0.740
Ramachandran							
favored (%)	97.65	92.91	94.86	95.07	95.08	94.24	96.92
allowed (%)	1.92	6.23	4.71	4.47	4.27	5.11	3.08
outlier (%)	0.77	0.86	0.43	0.47	0.65	0.65	0.00

*Values in parentheses are for highest-resolution shell.

Supplementary Tab. 9: Synthetic *P. aeruginosa* PA14/PAO1 RhlR 21-mer peptides for SPOT array (Supplementary Fig. 3a)

Position	A*	B	C	D
1	MRNDGGFLLWWDGLRSEMQPI	QMQNYGAVDPAILNGLRSSEM	SFEREEIRLRLRCMIELLTQK	KNIQKKFDAPNKTLAAAYAAA
2	DGGFLLWWDGLRSEMQPIHDS	NYGAVDPAILNGLRSSEMVVW	REEIRLRLRCMIELLTQKLTD	QKKFDAPNKTLAAAYAAALGL
3	FLLWWDGLRSEMQPIHDSQGV	AVDPAILNGLRSSEMVVWSDS	IRLRLRCMIELLTQKLTDLEH	KKFDAPNKTLAAAYAAALGLI
4	WWDGLRSEMQPIHDSQGVFAV	PAILNGLRSSEMVVWSDSLFD	RLRCMIELLTQKLTDLEHPML	
5	GLRSEMQPIHDSQGVFAVLEK	LNGLRSSEMVVWSDSLFDQSR	CMIELLTQKLTDLEHPMLMSN	
6	SEMQPIHDSQGVFAVLEKEVR	LRSSEMVVWSDSLFDQSRMLW	ELLTQKLTDLEHPMLMSNPVC	
7	QPIHDSQGVFAVLEKEVRRLG	SEMVVWSDSLFDQSRMLWNEA	TQKLTDLEHPMLMSNPVCLSH	
8	HDSQGVFAVLEKEVRRLGFDY	VVWSDSLFDQSRMLWNEARDW	LTDLEHPMLMSNPVCLSHRER	
9	QGVFAVLEKEVRRLGFDYYAY	SDSLFDQSRMLWNEARDWGLC	LEHPMLMSNPVCLSHREREIL	
10	FAVLEKEVRRLGFDYYAYGVR	LFDQSRMLWNEARDWGLCVGA	PMLMSNPVCLSHREREILQWT	
11	LEKEVRRLGFDYYAYGVRHTI	QSRMLWNEARDWGLCVGATLP	MSNPVCLSHREREILQWTADG	
12	EVRRLGFDYYAYGVRHTIPFT	MLWNEARDWGLCVGATLPIRA	PVCLSHREREILQWTADGKSS	
13	RLGFDYYAYGVRHTIPFTRPK	NEARDWGLCVGATLPIRAPNN	LSHREREILQWTADGKSSGEI	
14	FDYYAYGVRHTIPFTRPKTEV	RDWGLCVGATLPIRAPNNLLS	REREILQWTADGKSSGEIAII	
15	YAYGVRHTIPFTRPKTEVHGT	GLCVGATLPIRAPNNLLSVLS	EILQWTADGKSSGEIAIILSI	
16	GVRHTIPFTRPKTEVHGTYPK	VGATLPIRAPNNLLSVLSVAR	QWTADGKSSGEIAIILSISES	
17	HTIPFTRPKTEVHGTYPKAWL	TLPIRAPNNLLSVLSVARDQQ	ADGKSSGEIAIILSISESTVN	
18	PFTRPKTEVHGTYPKAWLERY	IRAPNNLLSVLSVARDQQNIS	KSSGEIAIILSISESTVNFHH	
19	RPKTEVHGTYPKAWLERYQMQ	PNNLLSVLSVARDQQNISSFE	GEIAIILSISESTVNFHHKNI	
20	TEVHGTYPKAWLERYQMQNYG	LLSVLSVARDQQNISSFEREE	AIILSISESTVNFHHKNIQKK	
21	HGTYPKAWLERYQMQNYGAVD	VLSVARDQQNISSFEREEIRL	LSISESTVNFHHKNIQKKFDA	
22	YPKAWLERYQMQNYGAVDPAI	VARDQQNISSFEREEIRLRLR	SESTVNFHHKNIQKKFDAPNK	
23	AWLERYQMQNYGAVDPAILNG	DQQNISSFEREEIRLRLRCMI	TVNFHHKNIQKKFDAPNKTLA	
24	ERYQMQNYGAVDPAILNGLRS	NISSFEREEIRLRLRCMIELL	FHHKNIQKKFDAPNKTLAAAY	

*Peptide A1 starts with residue 1 of RhlR, peptide D3 ends with the C-terminal residue 241. Note that the sequences of RhlR from the commonly used laboratory strains *P. aeruginosa* PA14 and PAO1 are identical (UniProt entries A0A0H2ZEG8 & P54292).

Source Data for Supplementary Figures (Uncropped photographs of western blots, SPOT arrays and gels)

Supplementary Figure 1a

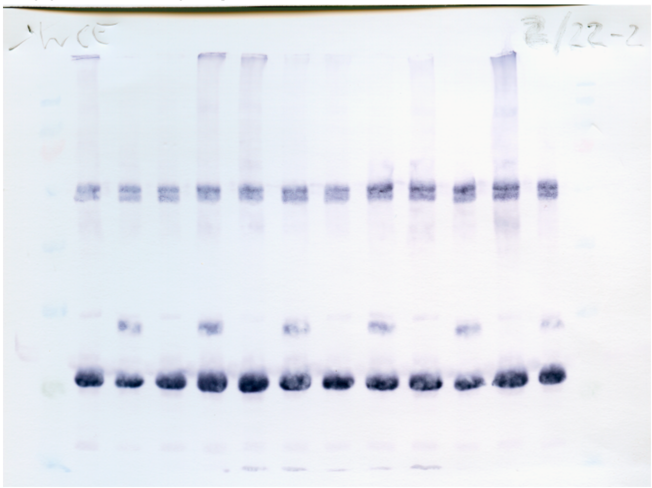

Supplementary Figure 2a

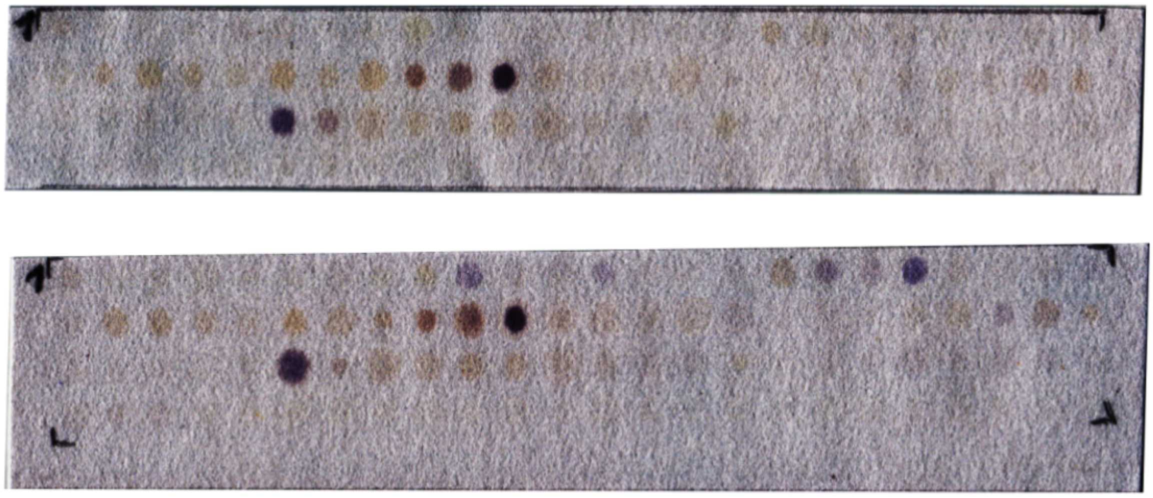

Supplementary Fig. 5d

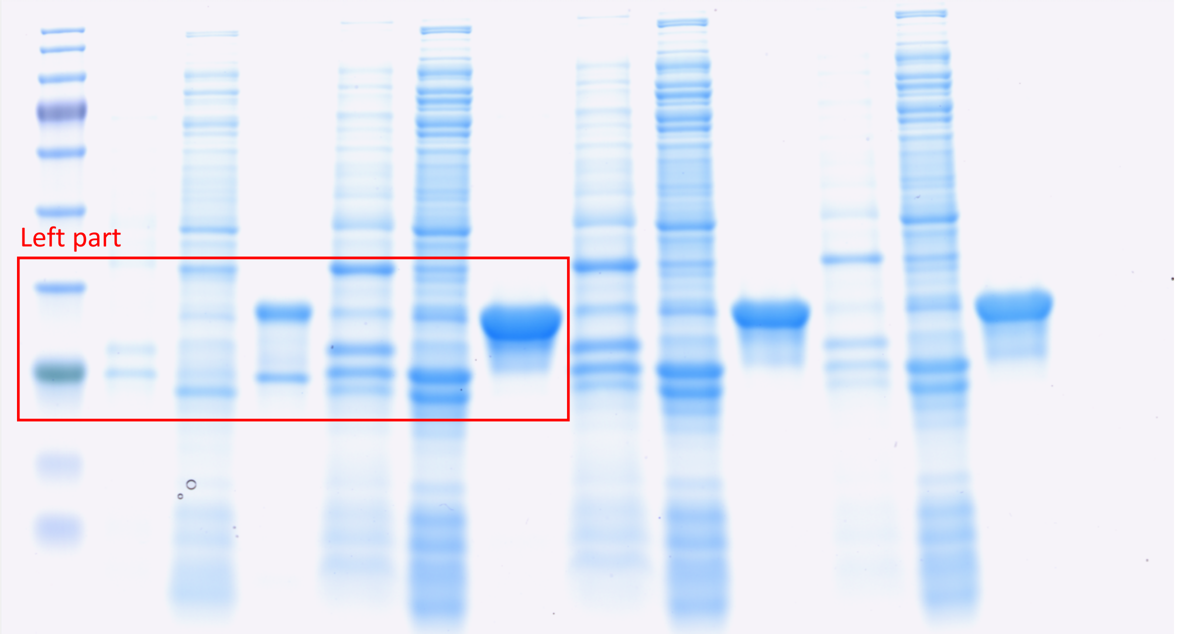

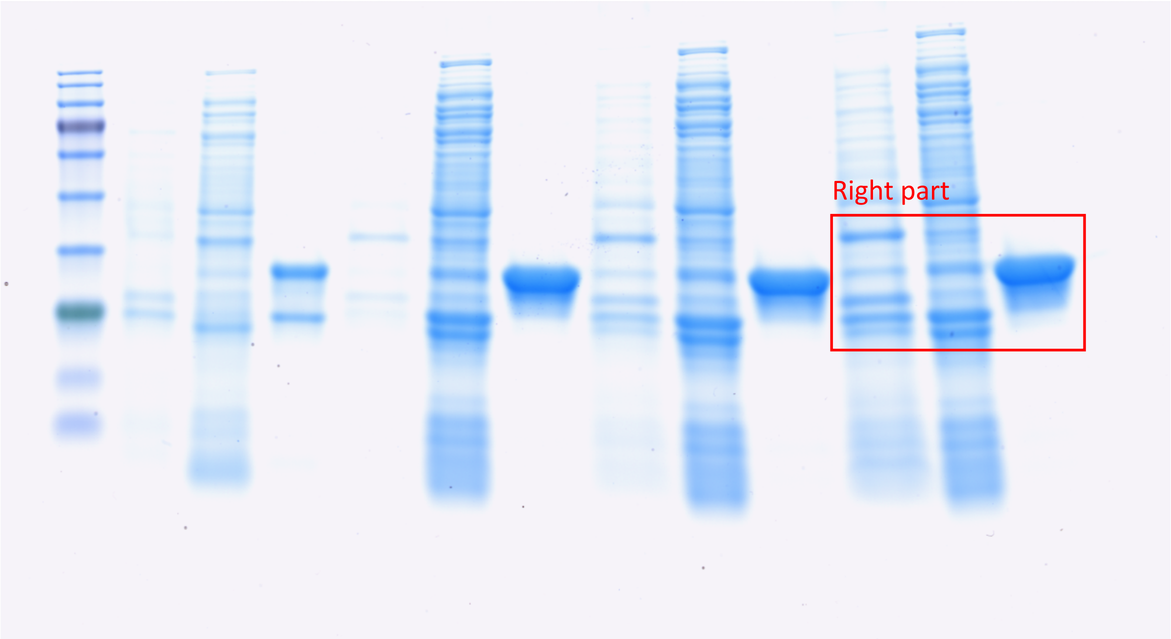

Supplement: Supplementary file 1 — Supplementary Information [file 41467_2022_35030_MOESM1_ESM.pdf]
