# Supplementary material for: Moonlighting chaperone activity of the enzyme PqsE contributes to RhlR-controlled virulence of Pseudomonas aeruginosa
Source: Nat Commun. 2022 Dec 1;13:7402. doi: 10.1038/s41467-022-35030-w (PMC9715718; doi:10.1038/s41467-022-35030-w)
Supplement: Supplementary file 3 — Reporting Summary [file 41467_2022_35030_MOESM3_ESM.pdf]

nature portfolio

Corresponding author(s): Wulf Blankenfeldt
Last updated by author(s): Nov 8, 2022

Reporting Summary

Nature Portfolio wishes to improve the reproducibility of the work that we publish. This form provides structure for consistency and transparency in reporting. For further information on Nature Portfolio policies, see our Editorial Policies and the Editorial Policy Checklist.

Statistics

For all statistical analyses, confirm that the following items are present in the figure legend, table legend, main text, or Methods section.

n/a	Confirmed	
☐	☒	The exact sample size (*n*) for each experimental group/condition, given as a discrete number and unit of measurement
☐	☒	A statement on whether measurements were taken from distinct samples or whether the same sample was measured repeatedly
☐	☒	The statistical test(s) used AND whether they are one- or two-sided<br>*Only common tests should be described solely by name; describe more complex techniques in the Methods section.*
☒	☐	A description of all covariates tested
☒	☐	A description of any assumptions or corrections, such as tests of normality and adjustment for multiple comparisons
☐	☒	A full description of the statistical parameters including central tendency (e.g. means) or other basic estimates (e.g. regression coefficient) AND variation (e.g. standard deviation) or associated estimates of uncertainty (e.g. confidence intervals)
☐	☒	For null hypothesis testing, the test statistic (e.g. *F*, *t*, *r*) with confidence intervals, effect sizes, degrees of freedom and *P* value noted<br>*Give P values as exact values whenever suitable.*
☒	☐	For Bayesian analysis, information on the choice of priors and Markov chain Monte Carlo settings
☒	☐	For hierarchical and complex designs, identification of the appropriate level for tests and full reporting of outcomes
☒	☐	Estimates of effect sizes (e.g. Cohen's *d*, Pearson's *r*), indicating how they were calculated

Our web collection on statistics for biologists contains articles on many of the points above.

Software and code

Policy information about availability of computer code

Data collection	Proteomic analysis of RhlR solubility in P. aeruginosa PA14: MSMS-method "DDA PASEF method for short gradients with 0.5 seconds cycletime" (Bruker);<br>Size-exclusion chromatography – multi-angle light scattering (SEC-MALS): Astra Version 7.3.2.21 (Wyatt Technology Corp.);<br>Microscalar Thermophoresis (MST): MO.control: v1.6.1 (NanoTemper Technologies GmbH);
Data analysis	Proteomic analysis of RhlR solubility in P. aeruginosa PA14: PaSER Version 2022c (Bruker), GraphPad Prism 9.3.1;<br>Identification of PqsE-interacting proteins in P. aeruginosa: MaxQuant Version 1.5.3.8;<br>Size-exclusion chromatography – multi-angle light scattering (SEC-MALS): Astra Version 7.3.2.21 (Wyatt Technology Corp.);<br>Microscalar Thermophoresis (MST): MO.Affinity Analysis v2.3 (NanoTemper Technologies GmbH);<br>Diffraction Data Indexing & Integration: XDS Versions Jan 2020 - Jan 2022,<br>Diffraction Data Scaling & Merging: AIMLESS (as included in ccp4 Version 7.1),<br>Anisotropic Diffraction Data Scaling & Merging: STARANISO server release v3.347,<br>Molecular Replacement: phaser (as included in phenix Versions 1.19 & 1.20),<br>Refinement of Macromolecular Structures: phenix.refine (as included in phenix Versions 1.19 & 1.20)<br>Interface Analysis of Macromolecular Structures: PISA (as included in ccp4 Version 7.1)

For manuscripts utilizing custom algorithms or software that are central to the research but not yet described in published literature, software must be made available to editors and reviewers. We strongly encourage code deposition in a community repository (e.g. GitHub). See the Nature Portfolio guidelines for submitting code & software for further information.

Data

Policy information about availability of data

All manuscripts must include a data availability statement. This statement should provide the following information, where applicable:

- Accession codes, unique identifiers, or web links for publicly available datasets
- A description of any restrictions on data availability
- For clinical datasets or third party data, please ensure that the statement adheres to our policy

Coordinates and diffraction data have been deposited in the Protein Data Bank with accession codes 7R3F [https://dx.doi.org/10.2210/pdb7R3F/pdb] (PqsE mutant E187A), 7R3E [https://dx.doi.org/10.2210/pdb7R3E/pdb] (PqsE-XTEN30-RhlR fusion protein in complex with mBTL), 8B4A [https://dx.doi.org/10.2210/pdb8B4A/pdb] (PqsE/RhlR:C4-HSL complex), 7R3J [https://dx.doi.org/10.2210/pdb7R3J/pdb] (PqsE/RhlR:mBTL complex), 7R3G [https://dx.doi.org/10.2210/pdb7R3G/pdb] (RhlR-P75 in complex with mBTL), 7R3H [https://dx.doi.org/10.2210/pdb7R3H/pdb] (RhlR-P75 in complex with C4-HSL) and 7R3I [https://dx.doi.org/10.2210/pdb7R3I/pdb] (RhlR-P61 in complex with mBTL).
Other protein structures used for analysis were obtained from the Protein Data Bank with accession codes 2UV0 [https://doi.org/10.2210/pdb2UV0/pdb] (Structure of the P. aeruginosa LasR ligand-binding domain bound to its autoinducer), 2Q0I [https://doi.org/10.2210/pdb2Q0I/pdb] (Structure of Pseudomonas Quinolone Signal Response Protein PqsE) and 6MWL [https://doi.org/10.2210/pdb6MWL/pdb] (LasR LBD:mBTL complex.
Mass spectrometry proteomics data have been deposited to the ProteomeXchange Consortium via the PRIDE partner repository with data set identifier PXD038000 [https://www.ebi.ac.uk/pride/archive/projects/PXD038000].
Source data are provided with this paper.

Human research participants

Policy information about studies involving human research participants and Sex and Gender in Research.

Reporting on sex and gender	n/a
Population characteristics	n/a
Recruitment	n/a
Ethics oversight	n/a

Note that full information on the approval of the study protocol must also be provided in the manuscript.

Field-specific reporting

Please select the one below that is the best fit for your research. If you are not sure, read the appropriate sections before making your selection.

☒ Life sciences ☐ Behavioural & social sciences ☐ Ecological, evolutionary & environmental sciences

For a reference copy of the document with all sections, see nature.com/documents/nr-reporting-summary-flat.pdf

Life sciences study design

All studies must disclose on these points even when the disclosure is negative.

Sample size	Experiments for quantitative analysis were performed in triplicates. Diffraction data were collected from single crystals for each reported data set and care was taken to collect highly redundant data as is the accepted standard in this research field.
Data exclusions	No data were excluded from analysis in experiments aiming at measuring molecular mass by size-exclusion chromatography/multi-angle light scattering, proteomic analysis by mass spectrometry, quantification of pyocyanin, protein melting point determination or measuring dissociation constants .<br>Resolution shells with an average I/sigma(I) value below 1.5 were excluded from model building and refinement in crystal structure analysis.
Replication	Experiments for quantitative analysis were performed with independent biological replicates (all experiments with bacteria) or as independent technical replicates (experiments with purified proteins, microscalar thermophoresis). Experiments with more qualitative character (gel electrophoresis, western blots) were performed with independent samples at least three times in the course of this work.
Randomization	Randomization was not performed since samples within each experimental set were generated and measured under identical conditions.
Blinding	Blinding was not required for this study because none of the included experiments relies on subjective interpretation.

Reporting for specific materials, systems and methods

We require information from authors about some types of materials, experimental systems and methods used in many studies. Here, indicate whether each material, system or method listed is relevant to your study. If you are not sure if a list item applies to your research, read the appropriate section before selecting a response.

Materials & experimental systems

n/a	Involved in the study
☐	☒ Antibodies
☒	☐ Eukaryotic cell lines
☒	☐ Palaeontology and archaeology
☒	☐ Animals and other organisms
☒	☐ Clinical data
☒	☐ Dual use research of concern

Methods

n/a	Involved in the study
☒	☐ ChIP-seq
☒	☐ Flow cytometry
☒	☐ MRI-based neuroimaging

Antibodies

Antibodies used

anti-RhlR polyclonal rabbit IgG (raised against recombinant RhlR:mBTL; Davids Biotechnology GmbH);
anti-PqsE polyclonal rabbit IgG (raised against recombinant PqsE; BioGenes GmbH);
anti-groEL polyclonal rabbit IgG (Abcam ab90522, Lot GR3394934-8);
anti-rabbit polyclonal goat IgG, AP-conjugated (Promega S3731, Lot 000515550);
anti-His6 monoclonal mouse IgG, AP-conjugated (Genetex GTX44021, Lot 821602997; cell line AD1.1.10)

Validation

anti-RhlR and anti-PqsE antibodies were validated inhouse with the respective recombinant proteins and with cell-lysates of recombinant E. coli bacteria overproducing these proteins from plasmid vectors.

All other antibodies are commercially available and have been validated by the respective suppliers. Their validation data are available on the manufacturers websites, as listed below:
https://www.abcam.com/groel-antibody-ab90522.pdf
https://www.promega.de/products/protein-detection/primary-and-secondary-antibodies/anti-rabbit-igg-fc-ap-conjugate/?catNum=S3731
https://www.genetex.com/PDF/Download?catno=GTX44021
